# A new technique for supracervical hysterectomy: Anterograde vaginal subtotal hysterectomy

**DOI:** 10.1097/MD.0000000000020006

**Published:** 2020-05-22

**Authors:** Zi-Jun Li, Zhen-Xiang Jia, Ya-Qin Zheng

**Affiliations:** a Department of Gynecology, ZheJiang QuHua Hospital, QuZhou, ZheJiang; b Department of Gynecology, Women and Children Hospital of TaiAn, TaiAn, ShanDong, China.

**Keywords:** anterograde transvaginal subtotal hysterectomy, hysterectomy, laparoscopic subtotal hysterectomy, non-prolapsed uterus, transvaginal natural orifice transluminal endoscopic surgery

## Abstract

To review the results of a novel method of subtotal hysterectomy, called anterograde vaginal subtotal hysterectomy (AVSH), and to compare them with those of laparoscopic subtotal hysterectomy (LSH).

We recruited 100 women with non-prolapsed uteruses and benign lesions of the uterus who required surgery. Of these, 60 underwent AVSH and 40 underwent LSH. Clinical data included average operation time, average volume of bleeding, postoperative anal exsufflation time, operative complications, average length of hospital stay and average hospital maintenance fee.

There were no significant differences in terms of average operation time, average length of hospital stay, or operative complications between the AVSH and LSH groups. The AVSH group showed early postoperative anal exsufflation (*P* = .000), and had a low average hospital maintenance fee (*P* = .000). The AVSH group showed a higher perioperative bleeding volume than the LSH group (*P* = .001), which may be a result of the relatively amateur AVSH technique.

AVSH is a minimally invasive, safe and feasible surgical procedure, with favorable early postoperative anal exsufflation and a low average hospital maintenance fee.

## 1 Introduction

Hysterectomy is a common gynecological procedure, performed in women with benign uterine conditions such as myoma, adenomyosis, or abnormal uterine bleeding (AUB). Hysterectomy is traditionally classified into 6 main categories: abdominal total hysterectomy, abdominal subtotal hysterectomy, vaginal total hysterectomy (VTH), laparoscopic total hysterectomy, laparoscopic subtotal hysterectomy (LSH), and laparoscopic-assisted total hysterectomy.^[1]^ The minimally invasive surgical method of choice for subtotal hysterectomy has been LSH.^[2–4]^ However, LSH has some disadvantages, for example, the risk of tumor cell dissemination caused by leiomyoma pulverization.^[5,6]^ For this reason, many gynecologists adopted laparoscopy-assisted vaginal subtotal hysterectomy (LAVSH) and transvaginal natural orifice transluminal endoscopic surgery (v-NOTES);^[8]^ however, both are relatively expensive variations on the conventional approaches to minimally invasive surgical approaches for vaginal subtotal hysterectomy (VSH) have not shown great improvement.^[7]^ Ten years ago, a gynecologist named Jia Zhen-Xiang pioneered anterograde VTH (AVTH) also called “Jia's vaginal surgery” in China. This study describes the new surgical technique and reports on the safety and feasibility of anterograde VSH (AVSH) in comparison with standard minimally invasive surgery techniques for laparoscopic subtotal hysterectomy (LSH).

## 2 Materials and methods

Patients: Between January 2015 to September 2017, 60 patients underwent AVSH in our hospital. There were 38 cases of uterine myoma, 12 cases of adenomyosis and 10 cases of adenomyosis complicated myoma. These were enrolled in the AVSH group. Of the patients who had LSH in the five years from January 2012 to January 2017, 40 were enrolled in the study. There were 28 cases of uterine myoma, 10 cases of adenomyosis and 2 cases of adenomyoma with leiomyoma.

Patients were eligible for inclusion if they had a history of vaginal delivery, a non-prolapsed uterus, uterine size not greater than a 12-week gravid uterus, normal uterus on pelvic examination, normal Thinprep Cytologic Test and high-risk HPV test, no evidence of endometrial malignant lesion (confirmed by pelvic ultrasonography, uterine curettage and hysteroscopy if there is suspicion of malignant transformation), no history of gynecological surgery. Informed consent was obtained from all patients. This study was approved by the ethics committee of the Zhe Jiang QuHua hospital. Clinical data collected include operation time, volume of bleeding, postoperative anal exsufflation time, the incidence rate of postoperative complications, length of hospital stay and average hospital maintenance fee. The hospital maintenance fee comprised operation costs, hospital bed fees, drug costs, medical consumable costs, nursing expenses, pathology, and anesthetic costs. The preoperative preparation was 3-days of vaginal washing with povidone-iodine solution and routine bowel preparation.

### 2.1 The key operative steps of AVSH

#### 2.1.1 Step 1

To expose the cervix (Fig. 1.1) a semi-annular incision between 0.5 and 0.8 cm is made in the anterior fornix of the vagina below the transverse sulcus of the bladder (Fig. 1.2). The uterovesical peritoneal reflection is exposed and excised to separate the vesicocervical space (Fig. 1.3).

**Figure 1:**
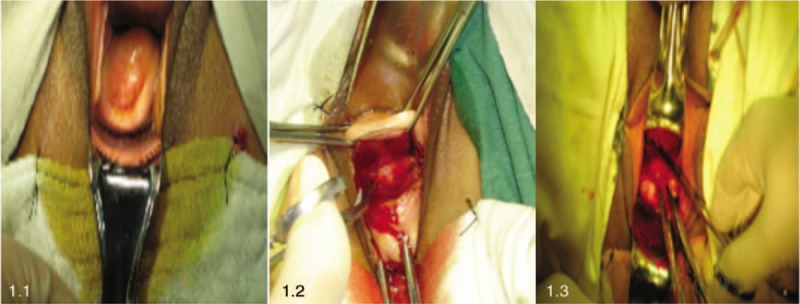
To open the anterior fornix of vagina and retroperitoneal bladder reflexion through vagina.

#### 2.1.2 Step 2

A myoma screw drill is used to flip the corpus uteri (Figs. 2.1–2.3) and expose 1 or both of the uterine cornua. For a large uterus, myomectomy, dissection of fibroids or the corpora uteri is first performed using a screw drill (Figs. 2.4–2.6) if the turn-over maneuver proves difficult.

**Figure 2:**
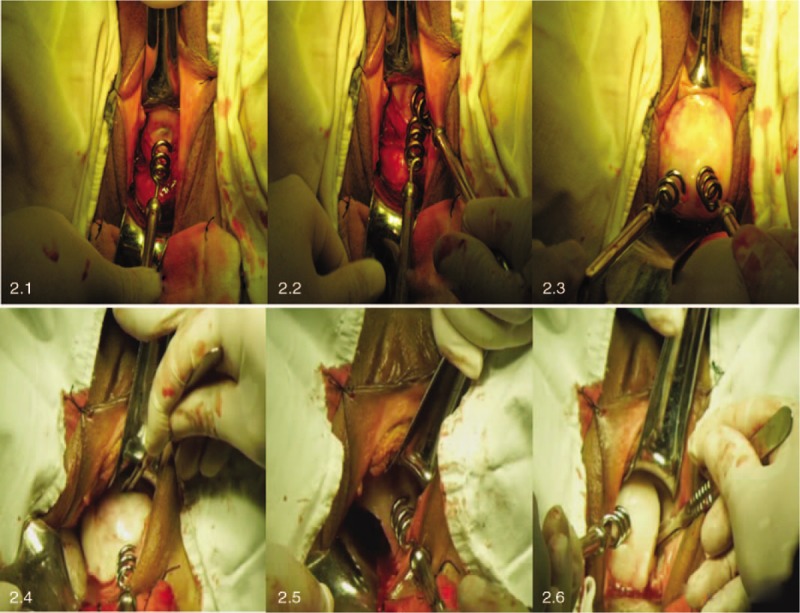
To flip the corpus uteri (Fig. 2.1–2.3) and dissect fibroids or the corpora uteri (Fig. 2.4–2.6) if the turn-over maneuver proves difficult.

#### 2.1.3 Step 3

**Figure 3:**
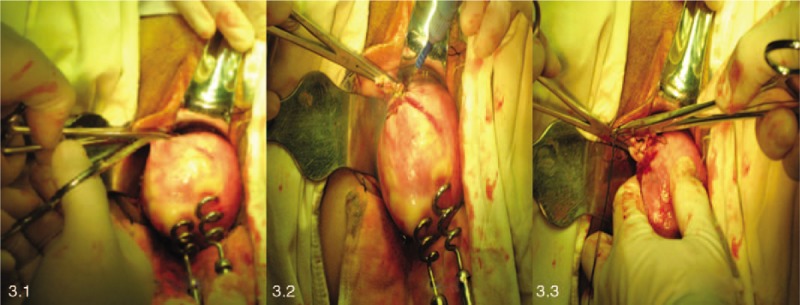
To manage the right uterine ligament, fallopian tube and uterine artery and vein.

The uterine ligaments of the right cornua uteri including the round ligament, the isthmic portion of the fallopian tube, the uterine ligament and the uterine arteriovenous supply are reflected upward (Fig. 3).

#### 2.1.4 Step 4

**Figure 4:**
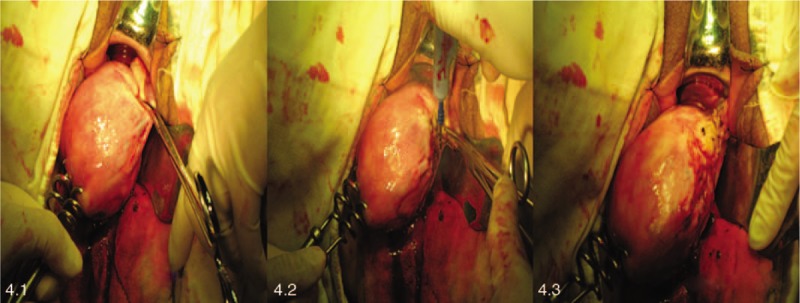
To manage the left uterine ligament, fallopian tube and uterine artery and vein.

The same process as step 3 is performed on the left (Fig. 4).

### 2.2 The surgical evaluation index

The data collected included operation time (min), volume of bleeding (mL), postoperative anal exsufflation time (hours), average length of hospital stay (days), average hospital maintenance fee (RMB) and the incidence of operative complications.

### 2.3 Statistical analysis

SPSS version 19.0 (SPSS Inc, Chicago, IL) was used for statistical analysis. General characteristics were analyzed and presented in means ± standard deviation (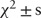) and the incidence of operative complications (%), using the paired-samples test and Pearson Chi-Square Test. A *P*-value of <.05 was considered significant.

## 3 Results

In terms of baseline data, differences between the AVSH group and LSH group in terms of age, body mass index, menopausal state and size of the uterus were not statistically significant. The groups were therefore comparable (Table 1).

**Table 1:**
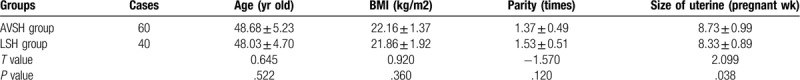
Comparison of general situations between 2 groups 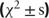.

Comparison of the operation evaluation indicators showed no significant differences between the groups in terms of average operation time (min), average length of hospital stay and the overall incidence of operative complications (*P* < .05). However, the AVSH group had a higher average volume of bleeding (*t* = -3.585, *P* = .001), early postoperative anal exsufflation time (*t* = -7.706, *P* = .000) and a lower average hospital maintenance fee (*t* = -21.372, *P* = .000), than did LSH (Table 2).

**Table 2:**
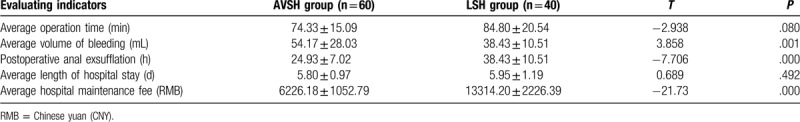
The comparison of operation evaluation indicators between 2 groups 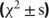.

The incidence of operative complications in the AVSH and LSH groups showed bladder injury in 1 case and 0, respectively, postoperative fever in 3 and 5 cases, respectively, stump hemorrhage and hematoma in 1 and 3 cases, respectively, and injury to the vaginal wall in 2 and 0 cases, respectively (Table 3). The overall complications did not differ between AVSH and LSH groups (*x*^*2*^ = 0.154, *P* = .695).

**Table 3:**
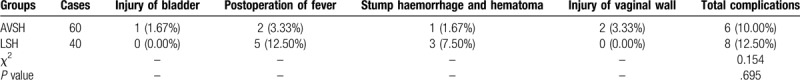
The comparison of operative complications between 2 groups (n %).

## 4 Discussion

SVH was originally described by Doderlein and Kronig in 1906, but did not gain popularity until the early 1990 s with the introduction of laparoscopic surgery. Since gaining recognition, several groups have reported successful case series.^[9,10]^ Their experience showed that SVH was very feasible;^[10,11]^ however, due to the limits of vaginal space, SVH was only practical for uteri equivalent to 12 to 14 weeks of pregnancy in size.

The AVSH, based on the traditional surgical procedure of vaginal hysterectomy, may appear impractical to surgeons used to the conventional approaches. A Chinese gynecologist, Zhen-Xiang Jia, from ShangDong Province, developed an unconventional VTH technique called “anterograde vaginal total hysterectomy” (AVTH). The method gained popularity, especially in the central regions of China.^[12–13]^ In particular, it dealt with the key difficulty of uterine corpus “switchover” with a purpose-made instrument called “screw drill” based on the level principle (Figs. 2.1–2.3). It also easily reduces the uterine body using a “screw drill” for myomectomy or dissection of fibroids or the corpora uteri (Figs. 2.4–2.6) when the turn-over maneuver proves difficult because of large-sized uterus. It simplified the surgical procedure for VTH and it was a natural progression to apply this to subtotal hysterectomy.

LSH is a popular minimally-invasive technique with the advantages of low average volume of bleeding, early postoperative recovery, short anal exsufflation time, and a low postoperative complication rate compared to the traditional abdominal subtotal hysterectomy^[2,4]^ VTH and vaginal natural orifice transluminal endoscopic surgery are reasonable alternatives for benign uterine diseases.^[5,14]^ Numerous studies have found that VTH is superior to laparoscopic total hysterectomy and it is the preferred operation according to current guidelines.^[15,16]^ However, VSH has been infrequently studied with few available reports.^[10,11]^ The principal problem of VSH is the difficulty in mobilizing the entire uterus into the vaginal introitus, especially for large uteri or cases with fibroids. However, AVSH completely solves this problem, because it can easily dissection of corpora uteri (Figs. 2.4–2.6) using a “screw drill.”

This study is the first report of AVSH. We found that the AVSH group did not differ significantly from the LSH group in terms of operation time, average length of hospital stay and the overall incidence of operative complications. The AVSH group also experienced early postoperative anal exsufflation time (*P* = .000). This study, however, showed a high average volume of bleeding (*P* = .001) in the AVSH group than in the LSH group, in line with findings of previous studies.^[17]^ Further analysis suggests that the main cause may be relatively unskilled AVSH surgical technique that can be an issue when confronted with a large-sized uterus or uterine fibroids. The process of “flipping” of the corpus uteri can cause the laceration of the vaginal wall. AVSH is an ideal NOTES technique,^[14,6]^ consistent with the minimally invasive surgical approach of SVH. It avoids the risk of tumor cell dissemination caused by uterine comminution^[6,18,19]^ according to the no-tumor-cell-dissemination principle of surgery. This study also showed that AVSH is an economical and practical surgical procedure with lower average hospital maintenance fee (*P* = .000) than that of LSH.

The results of this study are consistent with results of other studies.^[20]^ Although the rate of total postoperative complications showed no significant differences between groups, the rate of cervical stump hematoma in the AVSH group was lower than that of the LSH group (1.67% versus 7.50%). This was possibly caused by differences in surgical approach between AVSH and LSH, with the former requiring cervical ligation and the latter, cervical suture. In theory, AVSH does not involve opening the pouch of Douglas, therefore the chance of injury of the rectum is very small. The risk of bleeding may be reduced and bladder damage may be increased in theory. Nevertheless, the incidence of bladder damage was very low, only 1.67%.

## 5 Conclusion

AVSH is a safe, feasible, economical, and practical surgical technique with early postoperative anal exsufflation time, low average hospital maintenance fee, and a low rate of cervical stump hematoma. There were no differences in average operation time, average length of hospital stay, and the overall incidence of operative complications, compared with LSH.

## Author contributions

Surgical and Medical Practices: ZhenXiang, Jia Pro, The Women and Children Hospital of TaiAn, ShanDong province, TaiAn City, China.

**Statistical analysis:** Zi-Jun Li, Ya-Qin Zheng.

**Literature Search and Writing:** Zi-Jun Li, Ya-Qin Zheng.

## References

[1] Drahonovsky J Haakova L Otcenasek M A prospective randomized comparison of vaginal hysterectomy, laparoscopically assisted vaginal hysterectomy, and total laparoscopic hysterectomy in women with benign uterine disease. Eur J Obstet Gynecol Reprod Biol 2010;148:172–6. 19926201 10.1016/j.ejogrb.2009.10.019

[2] Donne J Smets M Polet R laparoscopic supracervical (subtotal) hysterectomy. Zentralbl Gynakol 1995;117:629–32. 8585357

[3] Gimbel H Total or subtotal hysterectomy for benign uterine disease? A meta-analysis. Acta Obstet Gynecol Scand 2007;86:133–44. 17364274 10.1080/00016340601024716

[4] American Association of Gynecologic Laparoscopists. AAGL practice report: practice guidelines for laparoscopic subtotal/supracervical hysterectomy (LSH). J Minim Invasive Gnecol 2014;21:9–16. 10.1016/j.jmig.2013.08.001 23954691

[5] Lauren N Wood Juzar Jamnagerwalla Melissa A Markowitz Public Awareness of Uterine Power Morcellation Through US Food and Drug Administration Communications: Analysis of Google Trends Search Term Patterns. JMIR Public Health Surveill. 2018 Apr 26;4:e47 10.2196/publichealth.9913 PMC5945987 29699965

[6] Harris JA Swenson CW Uppal S Practice patterns and postoperative complications before and after US Food and Drug Administration safety communication on power morcellation. Am J Obstet Gynecol 2016;214:9801–13. 10.1016/j.ajog.2015.08.047 26314519

[7] Baekelandt JF De Mulder PA Le Roy I Transvaginal natural orifice transluminal endoscopic surgery (vNOTES) adnexectomy for benign pathology compared with laparosopic excision (NOTABLE): a protocol for a randomized controlled trial. BMJ Open 2018;8:e018059. 10.1136/bmjopen-2017-018059 PMC5780723 29326183

[8] Wang CJ Yuen LT Lee CL Laparoscopic-assisted vaginal subtotal hysterectomy. I Laparoendosc Adv Surg Tech A 2005;15:33–7. 10.1089/lap.2005.15.33 15772474

[9] Tseng LH Liang CC Chang SH Vaginal subtotal hysterectomy and sacrospinous ligament fixation for treatment of uterine prolapse. J Gynecol Surg 2005;18:27–31.

[10] Pelosi MA 3rd Pelosi MA Subtotal vaginal hysterectomy: a new role for an old procedure. J Am Assoc Gynecol Laparosc 1997;4:479–83. 9224584 10.1016/s1074-3804(05)80043-x

[11] Magos AL Bournas N Richardson RE Subtotal vaginal hysterectomy. Minim Invasive Ther 1995;4:91–7.

[12] Jia ZX Ma XQ Ren P Clinical analysis of 900 cases of anterograde transvaginal subtotal hysterectomy. J Proceeding of Clinical Medicine 2008;17 (8B):738–9.

[13] Xue XL Dang RF Li HR Discussion on the clinical application of anterograde vaginal hysterectomy. MCHCC 2011;26:768–9.

[14] Turner Lindsay C Shepherd Jonathan P Li Wang Hysterectomy surgery trends: a more accurate depiction of the last decade? Am J Obstet Gynecol 2013;208:2771–7. 10.1016/j.ajog.2013.01.022 PMC3610857 23333543

[15] Papadopoulos Michail S Tolikas Athanasios C Miliaras Dimosthenis E Hysterectomy-current methods and alternatives for benign indications. Obstet Gynecol Int 2010;01–10. 10.1155/2010/356740 PMC2926674 20798870

[16] Nirmala Duhan Current and emerging treatments for uterine myoma-an update. Int J Womens Health 2011;231–41. 21892334 10.2147/IJWH.S15710 PMC3163653

[17] Hobson DT Imudia AN Al-Safi ZA Comparative analysis of different laparoscopic hysterectomy procedures. Arch Gynecol Obstet 2012;285:1353–61. 22124531 10.1007/s00404-011-2140-2

[18] John A Harris Carolyn W Swenson Shitanshu Practice patterns and postoperative complications before and after food and drug administration safety communication on power morcellation. Am J Obstet Gynecol 2016;214:9801–13. 10.1016/j.ajog.2015.08.047 26314519

[19] Erika L Mowers Courtney S Lim Bethany Skinner Patients’ knowledge and perceptions of morcellation. JSLS 2017;21:01–9. 10.4293/JSLS.2017.00009 PMC5491802 28694681

[20] Morgan DM Kamdar NS Swenson CW Nationwide trends in the utilization of and payments for hysterectomy in the United States among commercially insured women. Am J Obstet Gynecol 2018;218:425.el–-e1-18. 29288067 10.1016/j.ajog.2017.12.218 PMC5931386

